# In Vitro Evaluation of the Effects of Tylosin on the Composition and Metabolism of Canine Fecal Microbiota

**DOI:** 10.3390/ani10010098

**Published:** 2020-01-08

**Authors:** Carlo Pinna, Carla Giuditta Vecchiato, Monica Grandi, Ludovica Maria Eugenia Mammi, Claudio Stefanelli, Giacomo Biagi

**Affiliations:** 1 Department of Veterinary Medical Sciences, University of Bologna, via Tolara di Sopra 50, 40064 Ozzano Emilia (BO), Italy; carlo.pinna2@unibo.it (C.P.); carla.vecchiato2@unibo.it (C.G.V.); ludovica.mammi@unibo.it (L.M.E.M.); giacomo.biagi@unibo.it (G.B.); 2 Department for Life Quality Studies, University of Bologna, Corso d’Augusto 237, 47921 Rimini, Italy; claudio.stefanelli@unibo.it

**Keywords:** antibiotic-responsive enteropathy, dog, fecal microbiota, prebiotics, tylosin

## Abstract

**Simple Summary:**

The antibiotic-responsive enteropathy is a common canine chronic disorder for which tylosin represents an effective widely used therapeutic option, although its mechanism of action, beyond the well-known antibacterial activity, is still unclear. Given the beneficial role of prebiotic substrates for gut health, positive outcomes deriving from the association of tylosin with some prebiotic oligosaccharides might be supposed. The present study investigated in vitro the effects of tylosin, alone or supplemented with fructooligosaccharides, galactooligosaccharides, or xylooligosaccharides, on the composition and activity of the fecal microbiota of healthy dogs. It was partially confirmed that the antibacterial effect of tylosin, given the reduction of some microbial populations and metabolites, e.g., volatile fatty acids. Interestingly, the association of tylosin with prebiotics revealed counteracting effects on some undesirable changes exerted by tylosin, e.g., the reduction of bacteria generally considered beneficial such as lactobacilli and *Clostridium* cluster XIVa as well as volatile fatty acids, i.e., microbial fermentative end-products that are recognized as essential for enterocytes homeostasis.

**Abstract:**

The present study investigated the in vitro effects of tylosin (TYL), alone or associated with prebiotics (PRE), on selected canine fecal parameters. Eight treatments were set up: control diet with no addition of substrates; TYL; Fructooligosaccharides (FOS); Galactooligosaccharides (GOS); Xylooligosaccharides (XOS); TYL + FOS; TYL + GOS; TYL + XOS. The flasks (five for treatment), containing a canine fecal suspension (prepared with the feces of healthy adult dogs) and the residue of an in vitro digested dry dog food, were incubated in an anaerobic chamber at 39 °C. TYL and PRE were added at a concentration of 0.2 and 1 g/L, respectively. Samples were collected after 6 and 24 h for analyses. PRE decreased pH values, iso-butyrate, and iso-valerate throughout the incubation; increased lactobacilli, cadaverine, and, tendentiously, total volatile fatty acids after 6 h; increased *n*-butyrate, putrescine, spermidine, and reduced spermine and *E. coli* after 24 h. TYL resulted in lower total volatile fatty acids and lactobacilli and higher *Clostridium* cluster I after 6 h and higher pH values, spermidine, and *E. coli* throughout the study. When associated with TYL, PRE counteracted some undesirable effects of the antibiotic such as the decrease of lactobacilli and *Clostridium* cluster XIVa at both 6 and 24 h. In the present study, TYL exhibited inhibitory effects on canine fecal microbiota partially counteracted by PRE supplementation.

## 1. Introduction

Among the chronic gastrointestinal disorders in the dog, the antibiotic responsive enteropathy (ARE) represents a very common pathology, which is typically manifested through chronic diarrhea. The diagnosis is “empiric”, since it is traditionally based on the remission of the clinical signs within a few days from the beginning of antibiotic therapy, usually after an ineffective nutritional intervention aimed to exclude dietary adverse food reactions [1]. Nevertheless, the use of antibiotics is not resolutive for the treatment of ARE, since diarrhea commonly reappears within a few months after the suspension of the therapy; consequently, most of the dogs need lifelong treatments to achieve the control of the disease [2,3,4]. Among the most recommended antibiotics, there is a consensus that tylosin (TYL) represents an excellent therapeutic option. In this regard, the term “tylosin responsive diarrhea” (TRD) has been introduced in veterinary medicine to emphasize the effectiveness of this drug in dogs affected by idiopathic recurrent diarrhea [2]. Tylosin, a macrolide antibiotic registered exclusively for veterinary use, exerts its antibacterial action by binding to 23S rRNA of the bacterial ribosomal 50S subunit, thus preventing protein synthesis predominantly in gram-positive bacteria [5]. Furthermore, anti-inflammatory effects through the modulation of the synthesis of several mediators and cytokines involved in the inflammatory process have been proposed for this antibiotic [6].

The specific mechanism of action of TYL in the canine gut environment has not been clarified yet. Previously, an increase of *Enterococcus* spp. was observed during TYL administration both in healthy [7] and in enteropathic dogs [8]. In these last studies, the authors hypothesized that the selective pressure on the intestinal microbiota eventually induced by the antibiotic could favor this potentially probiotic bacterial population (enterococci isolated from dogs have been described to be resistant to TYL [9,10]) that, consequently, may have a certain role in the discontinuation of the diarrhea. Nonetheless, a recent study investigating the effects of TYL on fecal microbiota of healthy dogs reported a decrease in microbial diversity indices and in the presence of commensal anaerobic bacteria such as *Fusobacterium* and *Faecalibacterium* spp. [11], generally considered to be beneficial in dogs and frequently depleted in canine gastrointestinal diseases [12].

Anyway, there is a paucity of studies investigating the effects of TYL on canine gut microbiota, especially concerning compounds deriving from the bacterial metabolism such as volatile fatty acids (VFA), ammonia and biogenic amines, which are known to be of crucial relevance in host–microbial interactions [13], and some of them (VFA in particular) represent important indices of gut health [14,15].

Tylosin is generally considered a safe drug in canine species [2] and no published studies have described side effects in dogs. Nevertheless, it represents a per os antibiotic and, as such, it may negatively affect the intestinal microbiota, increasing the risk of dysbiosis [16]. In this regard, an increase of potential pathogens such as *E. coli* and *Cl. perfringens*-like organisms has been observed in the jejunal brush samples of healthy dogs receiving TYL at therapeutic doses, inducing the supposition of a potential risk for canine gastrointestinal health [7]. In this context, the use of a dietary prebiotic supplementation might represent a useful strategy to prevent potentially negative antibiotic-induced changes on the intestinal microbiota, even though, in this regard, conflicting evidences can be found in the literature [17,18].

According to the most recent definition, the term “prebiotic” is “a selectively fermented ingredient that results in specific changes in the composition and/or activity of the gastrointestinal microbiota, thus conferring benefit(s) upon host health” [19]. Likewise, fructooligosaccharides (FOS), galactooligosaccharides (GOS), and xylooligosaccharides (XOS) represent dietary non-digestible oligosaccharides (NDO) that pass through the gastrointestinal tract and have been recognized to exert “prebiotic effects” in humans [20,21] and animals such as dogs [22,23,24].

The aim of the present study was to investigate the in vitro effects of TYL, alone or associated with three prebiotic substrates (PRE) (FOS, GOS, and XOS) on some canine fecal microbial populations and metabolites, hypothesizing a beneficial influence exerted by PRE aimed to contrast the possible undesirable effects induced by TYL on fecal microbiota. Actually, during the in vitro incubation, TYL displayed inhibitory effects on canine fecal microbiota, partially counteracted by PRE supplementation.

## 2. Materials and Methods

The present study was conducted at the Laboratory of Animal Production of the Department of Veterinary Medical Sciences, University of Bologna, Italy.

### 2.1. Experimental Set Up

Six healthy adult dogs (mixed breed; average body weight of 18 kg; age 4–6 years) were fed the same commercial dry diet for adult dogs (Stuzzy New Zealand & Australia Dry Line with venison, Agras Delic Spa, Italy) for 4 weeks before the collection of fresh fecal samples. The diet contained the following ingredients: corn, barley, dehydrated venison, potato protein, purified pork fat, dried beet pulp, sunflower oil, brewer’s yeast, dried chicory pulp, FOS, cod liver oil, dicalcium phosphate, potassium chloride, sodium chloride, herbs (dog rose, bearberry, blackcurrant, taraxacum, and thistle), and *Yucca schidigera*. The macronutrient composition of the diet (per kg on dry matter basis) was the following: crude protein (CP) 236 g, ether extract (EE) 125 g, crude ash (ash) 57.1 g, starch 389 g, and crude fiber (CF) 20.8 g.

The same dry food that was fed to the dogs used as fecal donors was digested in triplicate using the two-step procedure proposed by Biagi et al. [25]. After in vitro digestion, the undigested fraction was dried at 65 °C until a constant dry weight was obtained (18.5 g of undigested residue were obtained from 100 g of food dry matter [DM]) and its chemical composition per kg was the following: CP 173 g, EE 24.3 g, starch 38.7 g, ash 146 g, and CF 99.4 g.

After the 4-week feeding period, a sample of fresh feces was collected from each dog immediately after excretion, pooled and suspended at 10 g/L in prereduced Wilkins Chalgren anaerobe broth. The fecal suspension was used to inoculate (100 mL/L) a previously warmed (39 °C) and prereduced medium prepared according to Sunvold et al. [26]. Five 30 mL bottles (each bottle containing 21 mL of fecal culture) were set up per treatment.

Eight treatments were carried out: (1) control diet with no addition of substrates; (2) Tylosin tartrate (TYL) (MP Biomedicals, Santa Ana, US); (3) FOS with a degree of polymerization between 2 and 8 (Beneo OPS, Beneo GmbH, Mannheim, Germany); (4) GOS (Vivinal GOS10, Friesland Foods Domo, Zwolle, the Netherlands; (5) XOS (Italfeed, Milano, Italy); (6) TYL + FOS; (7) TYL + GOS; (8) TYL + XOS. The bottles contained the in vitro digested commercial dry food for dogs at 10 g/L. PRE ingredients were added at the final concentration of 1 g/L. This dose should reflect the amount of PRE that reach the hindgut when they are included in a commercial extruded food for dogs (with a digestibility of approximately 90%) at a concentration of 10 g/kg. The TYL tartrate was added at 0.2 g/L, corresponding to the intestinal concentration of the antibiotic when orally administered at the daily dose of 25 mg/kg of body weight (according to Suchodolski et al. [7]) to a medium-sized dog (about 25 kg of body weight) receiving daily 300 g of extruded food (with a digestibility of approximately 90%). The pH of fecal cultures was adjusted to 6.7; bottles were sealed and incubated for 24 h at 39 °C in an anaerobic cabinet (Anaerobic System; Forma Scientific Co., Marietta, OH; under an 85% N_2_, 10% CO_2_ and 5% H_2_ atmosphere). Samples of fermentation fluid were collected from each bottle at 6 and 24 h for the determination of the pH, ammonia, biogenic amines, volatile fatty acids (VFA), and for microbial analysis.

### 2.2. Chemical Analyses

The commercial dry food and its undigested residue were analyzed according to the AOAC International standard methods (method 950.46 for water, method 954.01 for CP, method 920.39 for EE, method 920.40 for starch, method 942.05 for ash and method 962.09 for CF; AOAC) [27]. Ammonia was measured using a commercial kit (Urea/BUN—Color; BioSystems S.A., Spain). VFA were separated on a 2-m glass column (inner diameter, 3 mm) of 10% SP-1000 + 1% H_3_PO_4_ on 100/120 Chromosorb W AW with nitrogen as the carrier. The chromatograph was a Fisons HRGC MEGA 2 series 8560 with a flame ionization detector. The temperatures of the injector and detector were 200 °C, and the oven temperature was 155 °C. 2-ethylbutyric acid was used as the internal standard. For the determination of biogenic amines, samples were diluted 1:5 with perchloric acid (0.3 M); biogenic amines were later separated by HPLC and quantified through fluorimetry [28].

### 2.3. Microbial Analysis

At each sampling time, a 1 mL portion of fermentation fluid was collected from each vessel and centrifuged at 4 °C for 5 min, at 18,000× *g*. The supernatant was removed and immediately frozen at −80 °C for further analysis. Bacterial genomic DNA was extracted from remaining pellet using the Stool DNA isolation kit (Norgen Biotek Corp., Thorold, ON, Canada). Isolated DNA concentration (ng/μL) and purity were measured using a DeNovix DS-11 spectrophotometer (DeNovix Inc., Wilmington, DE, USA). Template DNA was diluted to 50 ng/μL and stored at −20 °C until further analysis. Total bacteria [29], *Escherichia coli* [30], *Bifidobacterium* and *Enterococcus* genera [31], *Lactobacillus* genus [32], *Clostridium* cluster I, and *Clostridium* cluster XIVa [33] were quantified via quantitative polymerase chain reaction (qPCR) using specific primers.

The qPCR assay was performed using a CFX96 Touch thermal cycler (Bio-Rad, Hercules, CA, USA).

Amplification was performed in duplicate for each bacterial group within each sample, while standard curves were run in triplicate.

Briefly, the PCR reaction contained 7.5 μL 2× SensiFAST No-ROX PCR MasterMix (Bioline GmbH, Luckenwalde, Germany), 4.8 μL of nuclease-free water, 0.6 μL of each 10 pmol primer and 1.5 μL of template DNA for a final reaction volume of 15 μL. The amplification cycle was as follows: initial denaturation at 95 °C for 2 min, 95 °C for 5 s, primer annealing at 56–64 °C for 10 s and 72 °C for 8 s. The cycle was repeated 40 times. A negative control (without the DNA template) was also run for each primer pair. Standard curves were constructed from eight tenfold dilutions for total bacteria, *Escherichia coli*, *Bifidobacterium* genus, *Lactobacillus* genus, *Enterococcus* genus, *Clostridium* cluster I, and *Clostridium* cluster XIVa. Cycle threshold values were plotted against standard curves for the quantification of the target bacterial DNA from fecal inoculum. Melting curves were checked after amplification to ensure the single product amplification of a consistent melting temperature.

### 2.4. Statistical Analyses

Data were analyzed by two-way ANOVA, with TYL and PRE as the main effects; the Newman–Keuls test was used as the post hoc test. When significant interactions (TYL x PRE) were found, individual means were analyzed by one-way ANOVA with Tukey’s test as the post hoc test. Five bottles (n = 5) were set up per treatment; each bottle represented an independent replicate. Significance and tendency for statistical tests were set at *p* < 0.05 and 0.05 < *p* < 0.1, respectively. Statistical analyses were performed using Statistica 10.0 software (Stat Soft Italia, Padua, Italy).

## 3. Results

The chemical parameters evaluated on the samples of fermentation fluid collected after 6 and 24 h of incubation are shown in Table 1 and Table 2, respectively. After 6 h of incubation, pH was increased by TYL (6.10 vs. 5.75 in flasks containing TYL and not, respectively; *p* < 0.001) and reduced by PRE (5.84 vs. 6.18 in flasks containing prebiotics and not, respectively; *p* < 0.001). Similarly, after 24 h of incubation, the pH was higher in treatments containing TYL (5.71 vs. 5.28 in flasks with TYL and not, respectively; *p* < 0.001) and decreased by PRE (5.43 vs. 5.69 in flasks containing PRE and not, respectively; *p* < 0.001). Moreover, the concentration of ammonia was reduced by both TYL and PRE after 6 h of incubation (−6.0% and −7.2%, respectively; *p* < 0.05).

During the present study, the concentrations of VFA were influenced by the treatments (Table 1 and Table 2). At 6 h, flasks containing TYL contained lower concentration of total VFA (−34%; *p* < 0.001). Conversely, PRE-treatments tended to increase this parameter, without reaching statistical significance (+2.1%; *p* = 0.085). Moreover, flasks with PRE contained higher concentrations of *n*-butyrate after 24 h of incubation (+24%; *p* < 0.001).

At 6 and 24 h significant interactions between PRE and TYL were observed in regard to acetate (*p* < 0.05), propionate (*p* < 0.05), iso-valerate and iso-butyrate (*p* < 0.001). After 6 h of incubation, acetate was reduced by all treatments except XOS. In particular, the association TYL + PRE reduced acetate more than FOS and GOS alone (Table 1). At 24 h, the decreasing effect on acetate was maintained only by TYL + PRE (*p* < 0.001) (Table 2). Both at 6 and 24 h, XOS annulled the decreasing effect exerted by TYL on propionate (*p* < 0.001). Moreover, throughout the study, both iso-acids were reduced by all treatments (except GOS at 6 h), most of all by TYL + PRE (*p* < 0.001) (Table 1 and Table 2).

In addition, significant interactions between PRE and TYL were observed in regard to *n*-butyrate at 6 h (*p* = 0.001) and total VFA at 24 h (*p* < 0.01). In particular, at 6 h PRE and TYL + GOS favored the increase of *n*-butyrate, differently from the other TYL + PRE that did not exert any effect (Table 1). At 24 h, XOS counteracted the decreasing effect exerted by TYL on total VFA (*p* < 0.05) (Table 2).

During the study, TYL and PRE treatments partially affected the concentrations of biogenic amines (Table 3). Putrescine was decreased by TYL after 6 h of incubation (−23%; *p* < 0.05) and increased both by TYL and PRE after 24 h (+30% and +7.7%, respectively; *p* < 0.001). Cadaverine was increased by PRE treatments after 6 h (+20%; *p* < 0.01). Furthermore, the interaction between PRE and TYL influenced this last parameter after 24 h of incubation (*p* < 0.01). In particular, GOS showed to counteract the increase of cadaverine produced by TYL (*p* < 0.05). Moreover, spermidine was increased by TYL both after 6 and 24 h (+137% and +54%, respectively; *p* < 0.05) and by PRE only at 24 h (+91%; *p* < 0.01). Finally, spermine resulted lower in flasks containing PRE after 24 h (−40%; *p* < 0.05).

**Figure 1:**
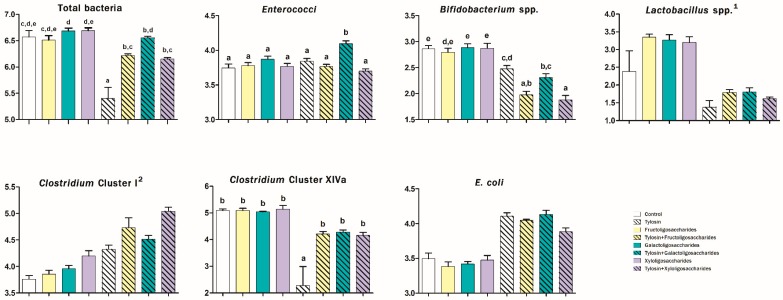
Microbial analysis (log_10_ copies DNA/ng DNA) after 6 h of an in vitro incubation of canine fecal inoculum with a control diet supplemented with some prebiotics and/or tylosin tartrate. Values are the means of five bottles per treatment. According to Two-way ANOVA (Newman-Keuls test as post hoc): ^1^ fructooligosaccharides, galactooligosaccharides and xylooligosaccharides differ from control (*p* < 0.05); ^2^ fructooligosaccharides differ from control (*p* < 0.05); xylooligosaccharides differ from control (*p* < 0.001). According to One-way ANOVA (Tukey’s test as post hoc): different letters indicate significant differences (*p* < 0.05).

**Figure 2:**
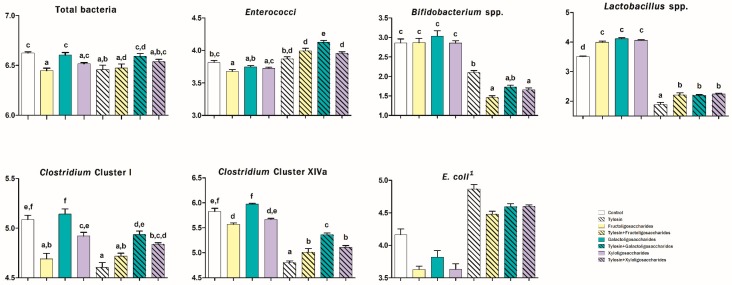
Microbial analysis (log_10_ copies DNA/ng DNA) after 24 h of an in vitro incubation of canine fecal inoculum with a control diet supplemented with some prebiotics and/or tylosin tartrate. Values are the means of five bottles per treatment. According to Two-way ANOVA (Newman-Keuls test as post hoc): ^1^ fructooligosaccharides, galactooligosaccharides and xylooligosaccharides differ from control (*p* < 0.001). According to One-way ANOVA (Tukey’s test as post hoc): different letters indicate significant differences (*p* < 0.05).

The abundances of some bacterial populations evaluated at 6 and 24 h of incubation in canine fecal inoculum are presented in Figure 1 and Figure 2, respectively.

After 6 h, treatments containing PRE increased the abundance of *Lactobacillus* spp. (2.51 vs. 1.88 log_10_ copies DNA/ng DNA; *p* < 0.05) and *Clostridium* cluster I (4.38 vs. 4.05 log_10_ copies/ng; *p* < 0.001) and reduced the presence of *E. coli* after 24 h (4.13 vs. 4.52 log_10_ copies/ng; *p* < 0.001).

TYL-treatments increased the abundance of *Clostridium* cluster I (4.65 vs. 3.95 log_10_ copies/ng; *p* < 0.001) and reduced the presence of lactobacilli (1.65 vs. 3.05 log_10_ copies/ng; *p* < 0.001) after 6 h. Furthermore, in both sampling times TYL increased the presence of *E. coli* (4.04 vs. 3.44 log_10_ copies/ng at 6 h and 4.64 vs. 3.81 log_10_ copies/ng at 24 h; *p* < 0.001). Significant interactions between PRE and TYL were observed in regard to the abundance of total bacteria (*p* < 0.01), enterococci (*p* < 0.01), bifidobacteria (*p* < 0.01), and *Clostridium* cluster XIVa (*p* ≤ 0.001) both at 6 and 24 h (Figure 1 and Figure 2) and in regard to the abundance of lactobacilli (*p* < 0.05) and *Clostridium* cluster I (*p* < 0.001) after 24 h of incubation (Figure 2). In particular, during the incubation, PRE counteracted the reduction of the abundance of total bacteria exerted by TYL. FOS, in particular, withdrew the TYL-effect only at 6 h while GOS at both 6 and 24 h (*p* < 0.05). TYL increased the presence of enterococci only when associated with PRE (only with GOS at 6 h and with all prebiotics at 24 h) (*p* < 0.05). Moreover, throughout the study, PRE did not display any effect on the abundance of bifidobacteria while treatments containing TYL favored their reduction, most of all when TYL was associated with FOS and XOS (*p* < 0.05) (Figure 1 and Figure 2). After 24 h, all of the PRE increased the presence of lactobacilli and counteracted the decreasing effect exerted by TYL on these bacteria (*p* < 0.001) (Figure 2). Moreover, FOS, TYL, their association, and TYL + XOS reduced the presence of *Clostridium* cluster I (*p* < 0.01). Finally, throughout the study, PRE counteracted the decreasing effect exerted by TYL on the abundance of *Clostridium* cluster XIVa (withdrawing it at 6 h) (*p* < 0.05) (Figure 1 and Figure 2).

## 4. Discussion

The purpose of this study was to evaluate the in vitro effects of TYL on some canine fecal microbial populations and metabolites and the possible influence resulting from the association with some prebiotic substrates (FOS, GOS, and XOS) commonly used in pet nutrition.

Previous studies based on 16S rRNA sequencing techniques have reported marked changes both in jejunal [7] and fecal [11] microbiota of healthy dogs after TYL treatment, in particular, with a significant decrease in mean bacterial diversity and a reduction of the relative abundance of the most important bacterial taxa.

Differently from the previous cited studies, the present investigation was based only on qPCR analysis and few specific bacterial groups have been evaluated. Consequently, the inhibitory effects observed in TYL-treatments have to be considered with caution. Indeed, throughout the in vitro incubation, the antibiotic decreased most of the bacteria analyzed, with the only exception of enterococci and *E. coli* (and *Clostridium* cluster I only at 6 h). Moreover, a significant reduction of VFA was observed, which could have favored the increase of pH measured in the same flasks. As VFA represent the most important metabolites derived from intestinal microbial activity [14], their substantial reduction might be a consequence of the microbial inhibition partially observed in TYL-treatments. TYL is known to exert its anti-bacterial effect especially on gram-positive bacteria [34]. Accordingly, in the present study, it was not surprising that in treatments containing this antibiotic the presence of lactobacilli, bifidobacteria and, partially, *Clostridium* spp. was reduced. Bifidobacteria and lactobacilli, in particular, are microbes generally considered as beneficial, since they are able to positively affect the intestinal environment, representing the main recognized genera of probiotics used in human [35] as well as in veterinary clinical practice [36]. Consequently, their reduction together with the decrease of some short chain fatty acids deriving from their metabolism (in particular, acetate and propionate) in TYL-treatments could be considered an undesirable outcome as well as the increase of the abundance of *E. coli* exerted by TYL throughout the incubation. *E. coli* is notoriously considered potentially negative for gastrointestinal health and its increase has been described among the microbial changes observed in dogs and cats affected by gastrointestinal diseases [12]. Anyway, the reduction of this bacterial species was expected since it is in accordance with other studies available in literature. For example, in a trial based on a mixed anaerobic continuous fermentation culture of chicken gastrointestinal microorganisms, TYL favored the persistence of *E. coli* 0157:H7 (together with a lower concentration of total VFA) while control cultures resulted in higher levels of VFA and showed to clear the pathogen [37]. Similarly, authors investigating the influence of TYL on the jejunal microbiota of healthy dogs described an increase of *E. coli* after the discontinuation of the therapy [7], supporting the hypothesis by which the modulation exerted by TYL on the intestinal microbiota might favor, in a direct or indirect way, the proliferation of particular gram-negative bacteria such as *E. coli*.

The knowledge deriving from the human literature concerning the effects of prebiotics on the intestinal ecosystem is well established. These substrates, in particular, have been widely described as non-digestible dietary components that, when reach the hindgut, support bacterial saccharolytic fermentations (in particular, through the stimulation of lactic acid bacteria (LAB) and bifidobacteria) [38], favoring VFA production (essential fuel for enterocytes and with a recognized immunomodulatory effect [14]) and, consequently, the acidification of the intestinal environment [17]. This last effect has been demonstrated to inhibit proteolytic microbial activity (with a consequent reduction of metabolites deriving from bacterial proteolysis such as ammonia and branched chain fatty acids (BCFA)) [38,39] and prevent the adhesion of pathogenic bacteria to the intestinal epithelium [40]. Accordingly, these commonly considered beneficial changes have been largely documented also in canine species, with particular regard to NDO [22]. Therefore, the positive changes observed during the present study in PRE-treatments (e.g., increased abundance of lactobacilli, higher concentrations of propionate and *n*-butyrate, the acidification of the pH, lower levels of BCFA together with the decrease of *E. coli* and a potentially toxic marker of bacterial proteolysis such as ammonia (even though only after 6 h) did not represent a surprising outcome. Nevertheless, FOS and XOS, when administered concurrently with TYL, favored a reduction of a notoriously beneficial microbial population such as bifidobacteria. In this regard, some authors pointed out that not all of the *Bifidobacterium* spp. are able to degrade oligosaccharides [20], as they are mainly specialized to metabolize complex carbohydrates, differently from lactobacilli that are known to ferment relatively simple mono- and oligo-saccharides such as FOS, GOS, or XOS [41]. Consistently with the present results, fecal bifidobacteria showed a tendency to decline in a previous trial carried out with healthy dogs receiving a dietary supplementation with FOS [42]. Regardless, the synergistic effect between TYL and PRE, such as FOS and XOS, appears difficult to explain.

Antibiotics can adversely affect the intestinal microbiota [43,44]. In this context, evidence supporting the efficacy of PRE to counteract the negative effects on the intestinal ecosystem deriving from antibiotic therapies has been reported by human literature [17,18]. During the present in vitro trial, the addition of PRE to the vessels containing TYL seemed to mitigate some of the effects exerted by this antibiotic that could be considered disadvantageous. In fact, the utilization of PRE resulted in the increase of total VFA (favored by XOS after 24 h), *n*-butyrate (favored by GOS after 6 h) and propionate (determined by XOS throughout the study) and higher abundance of lactobacilli (favored by all PRE at the end of the incubation). It might be supposed that these effects could be correlated since the metabolism of lactobacilli is based on the fermentation of non-digestible carbohydrates reaching the hindgut, mainly producing lactate [35]. In this regard, lactate (which has not been detected in the present study) is known to be rapidly converted in other VFA such as *n*-butyrate and propionate by intestinal microbiota [45]. Accordingly, during the present study, the presence of lactobacilli and the concentration of these VFA were increased in TYL-vessels when PRE were added.

In addition, also the counteracting effect favored by PRE on the TYL-induced decrease of the abundance of total bacteria and *Clostridium* cluster XIVa, can be positively considered. Interestingly, the reduction of total bacteria was completely withdrawn when GOS and FOS (at 6 h) and only GOS (at 24 h) were added to TYL as well as the decrease of *Clostridium* cluster XIVa by all PRE (only at 6 h). In particular, *Clostridium* cluster XIVa consists of commensal clostridia recognized as the main butyrate producers in human intestine [46]. Accordingly, during the incubation, the concentration of *n*-butyrate was increased by PRE, including when associated with TYL, except after 6 h when only GOS counteracted the TYL-induced reduction. The *n*-butyrate is known to be the preferred energy source for colonocytes, stimulate cell proliferation and apoptosis, and prevent colon cancer [47,48]. In this regard, some authors have previously emphasized the important role of *Clostridium* cluster XIVa in preserving gut health, describing a significant inhibition of these bacteria both in human and in canine chronic enteropathies [49].

Interestingly, the presence of TYL decreased the level of ammonia (only after the first 6 h of incubation) and BCFA (i.e., iso-butyrate and iso-valerate) and increased the concentration of biogenic amines, all metabolites deriving from bacterial proteolysis [50,51]. Moreover, also in PRE-treatments, higher levels of biogenic amines were observed throughout the study. If the reduction of the first two parameters in TYL-treatments might be related with the previously hypothesized antimicrobial effect exerted by the antibiotic, the increasing effect on several biogenic amines observed in both treatments containing TYL and PRE is more difficult to explain. The biological significance of biogenic amines still has not been clarified and several authors have proposed their beneficial influence on the intestinal mucosa [13]. In agreement with the present results, during previous in vitro studies with canine [52] or feline fecal inoculum [53], higher concentrations of biogenic amines (putrescine, in particular), were observed in vessels containing FOS. Moreover, in vivo trials with adult healthy dogs also displayed a linear increase of fecal putrescine, cadaverine, spermidine, and total amines after the dietary supplementation with increasing levels of FOS [54].

During the present study, a proteolytic and potentially pathogenic bacterial group, such as *Clostridium* cluster I, was unexpectedly increased by PRE and TYL after 6 h of incubation. Nevertheless, *Clostridium* cluster I, even if it is known to contain potentially pathogenic microbes, also includes commensal bacteria, which may contribute through their metabolic activity to gut homeostasis [55]. Interestingly, since clostridia are considered the main producers of biogenic amines [51], the increase of this clostridial cluster might be correlated with the higher levels of cadaverine and spermidine observed in the samples containing PRE and TYL, respectively. In agreement with the present study, the previously cited in vitro trial with feline fecal inoculum also showed an increase of *Cl. perfringens* (member of this last clostridial cluster) in FOS-vessels [53]. Regardless, these speculations do not explain the increase of biogenic amines observed at the end of the incubation, when clostridia were decreased or unaffected by treatments. In this regard, enterobacteria such as *E.coli*, and LAB such as lactobacilli (for which an increase was observed in TYL and PRE-treatments, respectively), have also been shown to produce these metabolites [51]. In particular, Spano et al. [56] reported that under acidic stress conditions several *Lactobacillus* spp. strains are able to produce biogenic amines. Therefore, it may be supposed that the increase of fermentation activity induced by PRE has resulted in lower pH values and lower concentrations of putrefactive metabolites such as ammonia and BCFA while the higher levels of biogenic amines may be partially attributable to the previously described acid tolerance mechanism expressed by lactobacilli. Concerning TYL-treatments, it might be speculated that the increase of biogenic amines might be the consequence of microbial modulation by the antibiotic. Given the recognized immunomodulatory effect of these last microbial metabolites [13], further studies would be warranted to investigate how TYL influence their production by fecal microbiota of enteropathic dogs.

Beyond the inhibitory effects shown by TYL, which do not help to clarify its efficacy in dogs affected by chronic diarrhea, the significant interaction observed between TYL and PRE concerning the abundance of *Enterococcus* spp. during the in vitro incubation is difficult to read. Surprisingly, enterococci were increased only when TYL was associated with PRE (with GOS, in particular). Conversely, PRE alone did not exert any effect (FOS even favored a decrease of these bacteria after 24 h). Since *Enterococcus* spp. have been reported to develop resistance to TYL [9,10,57], an increase of these bacteria favored by this antibiotic would have been expected in the present study, according to previous trials in the dog, where the proliferation of this potentially probiotic bacterial group was interpreted as a consequence of a potential selective pressure exerted by TYL on the intestinal microbiota both in healthy [7,11] and pathological conditions [8]. The opposite results highlighted during the present study induce to suppose that the response of enterococci towards the presence of TYL might have been influenced by the specific “in vitro” conditions here set up. Further research would be needed to investigate the apparent enhancement effect on enterococci observed when TYL and PRE were associated. Certainly, it should be emphasized that results here presented derive from an in vitro study that is not necessarily able to guarantee the exact in vivo conditions characterizing the canine hindgut environment. In particular, such a “closed” system precludes the evaluation of the physiological interactions occurring between host and intestinal microbiota. Consequently, the interpretations deriving from the present results should be cautiously considered.

## 5. Conclusions

During the present in vitro study, TYL displayed inhibitory effects on canine fecal microbiota. Interestingly, the supplementation with prebiotics such as FOS, GOS, and XOS showed to counteract some undesirable changes exerted by the antibiotic (in particular, the decrease of the abundance of recognized beneficial bacteria such as lactobacilli and *Clostridium* cluster XIVa). Nevertheless, this in vitro incubation was carried out by using fecal inoculum derived from healthy dogs, which might have harbored a stable and well-balanced microbiota. Further research aimed at investigating the influence of TYL, alone or associated with PRE, on the intestinal microbiota of enteropathic dogs is needed to clarify how this controversial antibiotic acts in the presence of intestinal dysbiosis.

## Figures and Tables

**Table 1:** pH values and concentrations (mmol/L) of ammonia and volatile fatty acids after 6 h of an in vitro incubation of canine fecal inoculum with a control diet supplemented with some prebiotics and/or tylosin tartrate ^1^.

	CTRL	FOS	GOS	XOS	TYL	TYL + FOS	TYL + GOS	TYL + XOS	ANOVA *p*-Value			Pooled SEM
									TYL × PRE	PRE	TYL	
pH	6.03	5.74	5.71	5.54	6.34	6.14	5.98	5.96	0.572	<0.001 ^2^	<0.001	0.061
Ammonia	27.9	23.2	26.8	25.7	25.2	24.0	24.6	23.6	0.302	0.028 ^3^	0.033	0.987
Acetic acid	12.4 ^d^	11.0 ^b,c^	10.3 ^b^	11.5 ^c,d^	6.58 ^a^	6.20 ^a^	6.13 ^a^	6.53 ^a^	0.020	<0.001	<0.001	0.249
Propionic acid	6.35 ^c,d^	7.17 ^e^	6.69 ^d,e^	8.04 ^f^	4.52 ^a^	4.90 ^a^	5.16 ^a,b^	5.72 ^b,c^	0.037	<0.001	<0.001	0.150
iso-Butyric acid	0.37 ^c^	0.23 ^b^	0.24 ^b^	0.27 ^b^	0.06 ^a^	0.05 ^a^	0.05 ^a^	0.04 ^a^	<0.001	<0.001	<0.001	0.010
*n*-Butyric acid	3.95 ^a^	4.95 ^d,e^	5.22 ^e^	4.39 ^b,c^	3.73 ^a^	4.01 ^a,b^	4.69 ^c,d^	3.96 ^a^	0.001	<0.001	<0.001	0.083
iso-Valeric acid	0.59 ^e^	0.36 ^c^	0.46 ^b^	0.42 ^d^	0.10 ^a^	0.09 ^a^	0.09 ^b^	0.10 ^a^	<0.001	<0.001	<0.001	0.009
Total VFA	23.9	23.7	22.9	24.7	15.0	15.2	16.1	16.4	0.148	0.085	<0.001	0.469

CTRL, control diet; FOS, fructooligosaccharides; GOS, galactooligosaccharides; PRE, prebiotics; TYL, tylosin; XOS, xylooligosaccharides. ^1^ Values are the means of five bottles per treatment. According to Two-way ANOVA (Newman-Keuls test as post hoc): ^2^ FOS differ from CTRL (*p* < 0.05); GOS differ from CTRL (*p* = 0.005); XOS differ from CTRL (*p* < 0.001). ^3^ FOS differ from CTRL (*p* < 0.05). According to One-way ANOVA (Tukey test’s as post hoc): means within a row with different letters differ (*p* < 0.05).

**Table 2:** pH values and concentrations (mmol/L) of ammonia and volatile fatty acids after 24 h of an in vitro incubation of canine fecal inoculum with a control diet supplemented with some prebiotics and/or tylosin tartrate ^1^.

	CTRL	FOS	GOS	XOS	TYL	TYL + FOS	TYL + GOS	TYL + XOS	ANOVA *p*-Value			Pooled SEM
									TYL × PRE	PRE	TYL	
pH	5.45	5.22	5.28	5.18	5.93	5.61	5.70	5.59	0.101	<0.001 ^2^	<0.001	0.019
Ammonia	36.9	35.1	36.0	34.6	36.0	34.8	35.7	36.1	0.268	0.136	0.958	1.255
Acetic acid	14.8 ^c^	14.2 ^c^	13.7 ^c^	13.9 ^c^	7.44 ^a,b^	7.12 ^a,b^	6.90 ^a^	8.37 ^b^	0.015	0.012	<0.001	0.284
Propionic acid	11.3 ^c^	12.0 ^c^	11.5 ^c^	12.2 ^c^	7.80 ^a^	9.75 ^b^	8.85 ^a,b^	11.8 ^c^	<0.001	<0.001	<0.001	0.270
iso-Butyric acid	0.53 ^e^	0.41 ^c^	0.46 ^d^	0.42 ^c^	0.07 ^b^	0.03 ^a^	0.03 ^a^	0.02 ^a^	<0.001	<0.001	<0.001	0.009
*n*-Butyric acid	4.82	6.12	6.37	5.31	4.65	5.97	6.16	5.34	0.708	<0.001 ^3^	0.121	0.112
iso-Valeric acid	0.83 ^c^	0.63 ^b^	0.78 ^c^	0.64 ^b^	0.14 ^a^	0.13 ^a^	0.12 ^a^	0.13 ^a^	<0.001	<0.001	<0.001	0.011
Total VFA	32.5 ^c^	33.7 ^c^	33.1 ^c^	32.8 ^c^	20.1 ^a^	23.0 ^a,b^	22.1 ^a^	25.6 ^b^	0.003	<0.001	<0.001	0.658

CTRL, control diet; FOS, fructooligosaccharides; GOS, galactooligosaccharides; PRE, prebiotics; TYL, tylosin; XOS, xylooligosaccharides. ^1^ Values are the means of five bottles per treatment. According to Two-way ANOVA (Newman-Keuls test as post hoc): ^2^ FOS, GOS e XOS differ from CTRL (*p* < 0.001); ^3^ FOS e GOS differ from CTRL (*p* < 0.001); XOS differ from CTRL (*p* < 0.05). According to One-way ANOVA (Tukey’s test as post hoc): means within a row with different letters differ (*p* < 0.05).

**Table 3:** Concentrations of biogenic amines (nmol/mL) after 6 and 24 h of an in vitro incubation of canine fecal inoculum with a control diet supplemented with some prebiotics and/or tylosin tartrate ^1^.

	CTRL	FOS	GOS	XOS	TYL	TYL + FOS	TYL + GOS	TYL + XOS	ANOVA *p*-Value			Pooled SEM
									TYL × PRE	PRE	TYL	
6 h												
Putrescine	150	144	164	179	103	161	91.8	136	0.341	0.478	0.046	25.0
Cadaverine	48.0	28.8	21.4	160	90.4	95.2	46.4	145	0.606	0.003 ^2^	0.178	30.6
Spermidine	41.0	19.5	14.2	15.3	50.4	58.6	29.0	75.0	0.170	0.191	0.001	12.3
Spermine	23.2	14.9	11.4	9.00	20.4	16.5	3.40	24.7	0.421	0.285	0.761	7.34
24 h												
Putrescine	152	161	189	157	206	207	233	210	0.671	<0.001 ^3^	<0.001	4.75
Cadaverine	52.2 ^a,b^	32.4 ^a^	55.6 ^a,b^	54.6 ^a,c^	117 ^d,e^	161 ^e^	102 ^b,c,d,f^	125 ^e,f^	0.009	0.476	<0.001	11.7
Spermidine	8.02	17.2	33.4	11.1	18.2	30.2	34.0	24.6	0.665	0.008 ^4^	0.030	5.82
Spermine	6.94	0.88	4.86	3.78	3.76	2.48	4.96	2.30	0.375	0.048 ^5^	0.462	1.41

CTRL, control diet; FOS, fructooligosaccharides; GOS, galactooligosaccharides; PRE, prebiotics; TYL, tylosin; XOS, xylooligosaccharides. ^1^ Values are the means of five bottles per treatment. According to Two-way ANOVA (Newman-Keuls test as post hoc): ^2^ XOS differ from CTRL (*p* < 0.01); ^3^ GOS differ from CTRL (*p* < 0.001); ^4^ GOS differ from CTRL (*p* < 0.01); ^5^ FOS tend to be different from CTRL (*p* = 0.06). According to One-way ANOVA (Tukey’s test as post hoc): means within a row with different letters differ (*p* < 0.05).
